# Clinical applications of transcranial magnetic stimulation in bipolar disorder

**DOI:** 10.1002/brb3.1419

**Published:** 2019-09-30

**Authors:** Alexandra K. Gold, Ana Claudia Ornelas, Patricia Cirillo, Marco Antonio Caldieraro, Antonio Egidio Nardi, Andrew A. Nierenberg, Gustavo Kinrys

**Affiliations:** ^1^ Department of Psychological and Brain Sciences Boston University Boston MA USA; ^2^ Outpatient Resistant Depression Clinic and Laboratory of Panic & Respiration Institute of Psychiatry Rio de Janeiro Brazil; ^3^ Serviço de Psiquiatria Hospital de Clínicas de Porto Alegre (HCPA) Porto Alegre Brazil; ^4^ Departamento de Psiquiatria e Medicina Legal Universidade Federal do Rio Grande do Sul Porto Alegre Brazil; ^5^ Department of Psychiatry Massachusetts General Hospital Boston MA USA; ^6^ Harvard Medical School Boston MA USA

**Keywords:** bipolar disorder, neuromodulation, transcranial magnetic stimulation

## Abstract

**Background:**

Many patients with bipolar disorder (BD) fail to experience benefit following traditional pharmacotherapy, necessitating alternative treatment options that will enable such patients to achieve remission. Transcranial magnetic stimulation (TMS) is a relatively new, noninvasive neuromodulation technique that involves the application of magnetic pulses on hyperactive or hypoactive cortical brain areas. We evaluated the existing literature on TMS as a treatment for BD across varied mood states.

**Methods:**

We searched PubMed up to October 2018 for original data articles published in English that evaluated outcomes in a bipolar sample across depressive, manic, mixed, and maintenance phases of BD.

**Results:**

Clinical trials of TMS for BD particularly suggest the potential of repetitive TMS for reducing depressive symptoms. Studies of TMS for mania have yielded more mixed findings. Few studies have evaluated TMS in other phases of the bipolar illness. TMS is generally associated with mild side effects though, in a few studies, it has been shown to contribute to a manic switch in previously depressed bipolar patients.

**Conclusions:**

Transcranial magnetic stimulation is a promising approach for treating patients with BD who have failed to respond to pharmacological or psychosocial treatment. Future research should more clearly elucidate which TMS protocols may be most effective for a given bipolar patient.

## Graphical abstract

We evaluated the existing literature on TMS as a treatment for bipolar disorder across varied mood states. Clinical trials of TMS for bipolar disorder particularly suggest the potential of repetitive TMS for reducing depressive symptoms, and TMS treatment is generally associated with mild side effects. Overall, TMS is a potentially promising treatment avenue for patients with bipolar disorder.

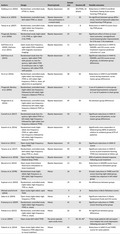

## 1 INTRODUCTION

Pharmacological agents have been effectively applied across all phases of the bipolar illness and, thus, are considered a first‐line treatment for bipolar disorder (BD; Fountoulakis et al., 2017). However, pharmacotherapy for BD has some notable limitations. Many patients with bipolar disorder fail to respond acutely to adequate pharmacotherapy (Geddes & Miklowitz, 2013). For those patients who do experience symptomatic improvements following pharmacological treatment, many experience frequent and intolerable side effects that lead to medication nonadherence and/or discontinuation (Matson et al., 2006; Shah, Grover, & Rao, 2017). In addition, several patients with BD suffer from an increased medical burden and clinicians must thus be mindful of interactions among the medications that patients could be taking to manage multiple medical concerns (Kemp et al., 2014; Martin, Williams, Haskard, & Dimatteo, 2005). To that end, the limitations of pharmacotherapy suggest the importance of alternative treatment options that will help patients with BD achieve and sustain remission (Martin et al., 2005).

Transcranial magnetic stimulation (TMS) is a relatively new, noninvasive therapeutic option that involves the application of magnetic pulses on hyperactive or hypoactive cortical brain areas with the aim of modulating brain networks (Brunelin et al., 2014). To administer TMS, the clinician places an electromagnetic coil on a prespecified region of the patient's scalp. Magnetic pulses from the coil travel through the skull toward a target cortical area, resulting in neural activation changes. To date, TMS has received the most consistent clinical and research application in treatment‐resistant depression (Connolly, Helmer, Cristancho, Cristancho, & O'Reardon, 2012; Loo & Mitchell, 2005). In the past several years, studies have explored the application of TMS in other psychiatric disorders. One initial randomized study in a combined unipolar and bipolar depressed sample evaluated daily TMS over the left prefrontal cortex relative to a sham treatment. Among the patients in this study, approximately 56% of responders had bipolar depression compared to approximately 44% of responders who had unipolar depression (George et al., 2000), supplying early evidence for the potential benefit of using TMS in a bipolar sample. Since that initial trial, other researchers have evaluated TMS for treating a range of mood symptoms in BD. The aim of this review is to explore the existing literature on the application of TMS across symptomatic and remitted stages of bipolar illness.

## 2 METHODS

We searched PubMed for relevant articles using the following search terms: (“Transcranial magnetic stimulation” AND “bipolar disorder”) (*n* = 181), (“TMS” AND “bipolar disorder”) (*n* = 65), (“Transcranial magnetic stimulation” AND “bipolar depression”) (*n* = 200), (“TMS” and “bipolar depression”) (*n* = 68), (“Transcranial magnetic stimulation” AND “mania”) (*n* = 201), (“TMS” and “mania”) (*n* = 74), (“Transcranial magnetic stimulation” AND “hypomania”) (*n* = 18), and (“TMS” AND “hypomania”) (*n* = 0). All search fields of the databases were included to maximize inclusivity. The research took place in October 2018, and no time restriction was placed on any of the database searches. Manual searches were also conducted using the reference lists from identified articles.

Eligible studies were original data articles exploring the application of TMS as a treatment strategy across various stages of a bipolar episode. Articles were not included if they combined unipolar and bipolar samples without separately evaluating outcomes in both disorders or if the specific TMS protocol was unclear. Only articles published in English in peer‐reviewed journals were eligible. Case studies with fewer than five patients, review papers, and theoretical articles were excluded. Results of the search were compared to exclude repeated references. Following this step, titles and abstracts were assessed to select potentially eligible articles. These articles were read in full to confirm they were relevant for the present review.

## 3 RESULTS

### 3.1 TMS in bipolar depression

Most studies evaluating the application of TMS in bipolar depression have focused on repetitive transcranial magnetic stimulation (rTMS) which involves repeated magnetic doses at a set intensity level to a specified brain area (Mishra et al., 2011). Two seminal rTMS studies in an exclusively bipolar sample yielded mixed results. Dolberg and colleagues conducted a randomized, controlled trial evaluating active rTMS (20 sessions) relative to a sham intervention (10 sessions) for bipolar depression (*n* = 20; Dolberg, Dannon, Schreiber, & Grunhaus, 2002). The authors found that the active group had statistically significant improvements in psychiatric outcomes as evaluated via the Hamilton Depression Rating Scale (HAM‐D; Hamilton, 1960) and Brief Psychiatric Rating Scale (Overall & Gorham, 1962) though, of note, improvements were most prominent after the first two weeks of treatment (Dolberg et al., 2002). In a second study, Nahas and colleagues randomly assigned patients (*n* = 23) with bipolar depression (with two participants in a mixed state; e.g., both depressive and manic) to receive 10 sessions of left prefrontal rTMS (5 Hz) or a sham treatment over a two‐week period. The prefrontal region was selected for TMS application given data from prior studies which found that consistent stimulation of prefrontal areas yielded mood benefits. Post‐treatment, though the protocol was well‐tolerated by participants, there were no significant differences between the groups in symptomatic improvements (there was a trend of improved subjective mood favoring the active group; Nahas, Kozel, Li, Anderson, & George, 2003). Results from these two initial trials were sufficiently promising to warrant subsequent intervention research though some important questions remained. First, could rTMS yield a consistently potent response relative to a sham treatment such that modulation of the specific target brain area produced symptomatic improvements (as opposed to the psychological impact of receiving what may or may not have been a neurological treatment)? Moreover, if rTMS is able to consistently yield important mood benefits in BD, is there a defined window for symptomatic improvements? In a subsequent randomized trial, Tamas, Menkes, and El‐Mallakh (2007) randomly assigned participants (*n* = 5) to receive 8 sessions (4 weeks) of low‐frequency (1 Hz) active rTMS or a sham treatment over the right DLPFC. In this study, the sham group consisted of a single participant, a not‐insignificant limitation. Participants receiving rTMS demonstrated greater improvements in depressive symptoms (as assessed via the HAM‐D; Hamilton, 1960) relative to those receiving a sham treatment, though the benefits favoring the rTMS group did not emerge until two weeks post‐treatment (Tamas et al., 2007), a duration that contrasts with the timeframe for improvements evidenced in the study conducted by Dolberg and colleagues (Dolberg et al., 2002). Ultimately, these data suggest it may be difficult to broadly apply a predetermined time frame of rTMS treatment or to expect treatment gains within a specific time period. Certain clinical variables may be associated with the need for a longer duration of rTMS treatment in BD (e.g., more than 15 rTMS sessions). Older patients with a longer, more refractory, and more severe bipolar depression may require more rTMS sessions than patients with a shorter, less chronic bipolar depression (Cohen, Brunoni, Boggio, & Fregni, 2010).

Since these initial studies, follow‐up clinical trials of rTMS in bipolar depression have focused on fine‐tuning the precision of administration through a focus on specific variables. Many studies of rTMS in unipolar and bipolar depressed samples have historically incorporated left‐sided dorsolateral prefrontal cortex (DLPFC) with high‐frequency rTMS. Data across several studies suggest particular benefits of this location/frequency combination for depression (Dolberg et al., 2002; Fitzgerald et al., 2016; Nahas et al., 2003). Some studies have also showed the benefit of rTMS applied at a low frequency over the right DLPFC. Fitzgerald and colleagues randomly assigned patients with treatment‐resistant depression (*n* = 25 patients with BD) to one of two low‐frequency right‐sided protocols (1 Hz vs. 2 Hz). Patients received 10 daily sessions followed by an additional 2 weeks (10 sessions) of rTMS if they showed an initial treatment response (based on HAM‐D (Hamilton, 1960) scores). Study findings revealed that time was significantly associated with improvements in depression with a trend of greater improvements for patients receiving the 2 Hz protocol relative to the 1 Hz protocol (Fitzgerald, Huntsman, Gunewardene, Kulkarni, & Daskalakis, 2006). Dell'Osso and colleagues evaluated the efficacy of low‐frequency (1 Hz) rTMS over the right DLPFC in patients (*n* = 11) with bipolar depression for a duration of 3 weeks (Bernardo Dell'Osso et al., 2009). This study was unique in that it combined rTMS with brain navigation, or use of magnetic resonance imaging to precisely target the most relevant cortical region for a given patient. Post‐treatment, patients demonstrated significant improvements in symptoms of depression (as assessed via the HAM‐D (Hamilton, 1960) and the Montgomery‐Asberg Depression Rating Scale [MADRS] (Montgomery & Asberg, 1979)) and reductions in overall illness severity (as assessed via the Clinical Global Impression scale [CGI]; Bernardo Dell'Osso et al., 2009; Spearing, Post, Leverich, Brandt, & Nolen, 1997). Following this acute trial, all participants were followed prospectively in a naturalistic study that assessed the long‐term effects of the rTMS treatment for a period up to 1 year (Dell'osso, D'Urso, Castellano, Ciabatti, & Altamura, 2011). Outcomes were assessed via monthly HAM‐D and Young Mania Rating Scale (YMRS; Young, Biggs, Ziegler, & Meyer, 1978) evaluations. Among the 11 patients, 4 patients maintained symptomatic improvement at 1‐year follow‐up. Notably, successful response to acute rTMS predicted sustained response at follow‐up whereas a lack of response to acute rTMS predicted nonresponse at follow‐up (Dell'osso et al., 2011).

Fewer studies have evaluated the comparative effectiveness of high‐ versus low‐frequency rTMS of the right versus left DLPFC in the context of a single study. In a subsequent trial, Dell'Osso et al. (2015) randomized patients (*n* = 33) to receive one of three 20‐session rTMS protocols over a four‐week period: (a) low‐frequency rTMS (1 Hz) over the right DLPFC incorporating pauses at specified points (420 stimuli per session), (b) low‐frequency rTMS (1 Hz) over the right DLPFC at a continuous rate (900 stimuli per session), or (c) high‐frequency rTMS (10 Hz) over the left DLPFC incorporating pauses at specified points (750 stimuli per session). At post‐treatment, patients demonstrated significant reductions in depression and illness severity outcomes (as assessed via the HAM‐D, MADRS, and CGI) with no significant group differences in treatment efficacy or tolerability (Dell'Osso et al., 2015). Thus, these data highlight flexibility in rTMS protocols such that patients may still be able to experience benefits regardless of the frequency or location of the rTMS treatment; a novel finding suggesting that the widely followed left DLPFC, high‐frequency rTMS protocol may not be the only effective option. Moreover, these data may indicate that patients who do not respond to a particular rTMS protocol could benefit from a different protocol (e.g., different frequency and/or cortical target). Notably, one study randomly assigned patients with bipolar II depression (*n* = 38) to receive 20 sessions of left high‐frequency rTMS, right low‐frequency rTMS, or a sham intervention over a four‐week period. All participants received adjunctive quetiapine. There were no significant differences among the three groups, indicating that TMS combined with quetiapine was not more effective than quetiapine combined with sham treatment and that there was no difference in outcomes between the two stimulation thresholds (Hu et al., 2016).

Studies have also suggested the benefit of sequentially applied bilateral rTMS stimulation for bipolar depression. An early study conducted by Fitzgerald and colleagues assessed right‐sided, low‐frequency (1 Hz) followed by left‐sided, high‐frequency (10 Hz) active rTMS compared to sham treatment for treatment‐resistant depression (Fitzgerald, Benitez, et al., 2006). Patients received 10 sessions of rTMS with additional sessions offered if they were showing a response to rTMS (assessed via MADRS scores) for a total maximum treatment period of 6 weeks. Though this study had an overall sample size of 50 patients, only eight patients had BD. The authors found that 2 out 4 patients in the active group, compared to 1 out of 4 in the sham group, demonstrated treatment response (Fitzgerald, Benitez, et al., 2006). The small sample of bipolar patients in this study makes it difficult to draw strong conclusions regarding the effectiveness of a bilateral rTMS approach for BD. However, Fitzgerald and colleagues subsequently evaluated 20 sessions of active, sequential bilateral (right‐sided, low‐frequency [1 Hz] followed by left‐sided, high‐frequency [10 Hz]) rTMS relative to sham treatment for a four‐week period among patients (*n* = 49) with bipolar depression (Fitzgerald et al., 2016). Post‐treatment, no significant differences were found between the two groups, suggesting that the bilateral approach to rTMS may not be more helpful for treating psychiatric symptoms in BD as the historically applied unilateral approach (Fitzgerald et al., 2016). More promising findings were found in a naturalistic study of bipolar patients (*n* = 50) who sought clinical care at a rTMS clinic, though this study is limited by a lack of a control group (Carnell, Clarke, Gill, & Galletly, 2017). Patients received 18 sessions of low‐frequency [1 Hz] right‐sided rTMS over the DLPFC (either a 15 or 30‐min protocol) or bilateral rTMS over the DLPFC (intermittent high frequency [10 Hz] over the left DLPFC followed by continuous low‐frequency [1 Hz] over the right DLPFC, 15 min on each side) within a 6‐week period. There were no differences in outcomes across the various protocols, with all patients showing significant improvements in depression scores from baseline to post‐treatment (as assessed by the HAM‐D; Hamilton, 1960). Notably, outcomes were similar between unipolar and depressed bipolar patients receiving rTMS at the center (Carnell et al., 2017). However, in a separate study evaluating 20 sessions of bilateral (right DLPFC, low‐frequency [1 Hz] followed by left DLPFC, high‐frequency [10 Hz]) versus unilateral (right‐sided DLPFC, low‐frequency [1 Hz]) rTMS for bipolar depression (*n* = 30), the proportion of rTMS responders was significantly greater in the bilateral group relative to the unilateral group (Kazemi et al., 2016). This study incorporated a unique outcome measure of beta wave activity (as measured via electroencephalography) on the basis of data suggesting that depression is associated with enhanced beta frequency oscillations in frontal and occipital brain areas (Kazemi et al., 2016; Özerdem, Güntekin, Tunca, & Başar, 2008). Indeed, post‐treatment, the authors found that responders to rTMS had significantly decreased beta frequency oscillation, a finding that highlights a possible biological marker for assessing response to rTMS (Kazemi et al., 2016).

Lastly, some studies have incorporated novel technology with the goal of enhancing the efficacy of rTMS protocols. One innovative approach involves modification of the coil used in standard TMS treatment. Many rTMS protocols incorporate a coil that provides restricted depth, thus potentially limiting the capacity of direct stimulation over the relevant cortical region. Some data suggest that a novel H1‐coil allows a magnetic field that can enable treatment to occur over a wider area and with greater depth of stimulation. In recent years, the H1‐coil has been the focus of a few clinical trials in BD. One initial study among patients with bipolar depression (*n* = 19) found that 20 sessions of high‐frequency (20 Hz) rTMS delivered through an H1‐coil (deep TMS) over a 4‐week period led to a significant decrease in HAM‐D scores (Hamilton, 1960; Harel et al., 2011). A subsequent, randomized controlled study evaluated 20 sessions of add‐on, high‐frequency (18 Hz) deep TMS over the left DLPFC versus a sham treatment for patients with treatment‐resistant bipolar depression (*n* = 50). Patients receiving the active treatment demonstrated significantly greater improvements in HAM‐D scores relative to patients in the sham group though these gains were not maintained at follow‐up (Tavares et al., 2017). Finally, an open trial that incorporated 20 patients with bipolar I depression evaluated 20 sessions of add‐on, high‐frequency (20 Hz) bilateral deep TMS over the DLPFC. On the basis of HAM‐D scores, 80% of patients with BD showed a response immediately following acute treatment with 75% showing a response at 6‐month follow‐up (Rapinesi et al., 2018). It will be important for subsequent deep TMS studies to clarify an optimal number of acute treatment sessions and to evaluate whether an additional phase of maintenance treatment can enhance outcomes (Tavares et al., 2017), along with systematically evaluating a unilateral versus bilateral stimulation approach.

More recently, a modified rTMS approach known as theta burst stimulation (TBS) has been applied to bipolar depression. Data suggest that TBS may exert faster, stronger, and more sustained effects than traditional rTMS protocols (Beynel et al., 2014; Bulteau et al., 2017). Beynel and colleagues evaluated three weeks of randomly assigned daily intermittent TBS (iTBS; involving administration of magnetic pulses in bursts, which is thought to contribute to longer‐lasting neural effects) or sham treatment in patients (*n* = 12) with bipolar depression (Beynel et al., 2014). This study incorporated an antisaccade (AS) task which was completed on the first day of each week before and after iTBS treatment. Patients were placed in a dark room in front of a computer screen and asked to fix their attention on a dot in the center of the screen. During AS trials, patients were instructed to look in specific directions upon exposure to different colored cues. At post‐treatment, patients receiving the active iTBS demonstrated improvements in depressed mood (as assessed via the MADRS; Montgomery & Asberg, 1979) with mood improvements correlated with antisaccade task performance; a finding that reflects the potential of the task to be used as a metric of response to TMS treatment. Collectively, data on enhancements to traditional rTMS protocols (e.g., H‐coil, iTBS) are promising and reflect future avenues for research.

### 3.2 TMS in mania

Relative to bipolar depression, TMS has been less extensively studied as a treatment during the manic phase, potentially due to concerns that TMS can induce a manic episode in some patients (refer to the Discussion for findings on manic switches in some bipolar patients following TMS). In addition, whereas most TMS studies in bipolar depression have focused on rTMS, approximately half of the studies in mania have centered on traditional TMS protocols. Finally, in studies of TMS for mania, nearly all protocols have targeted the right prefrontal region. This pattern stems from an early clinical trial conducted by Grisaru and colleagues in which manic patients (*n* = 16) were randomly assigned to 10 sessions of right prefrontal or left prefrontal high‐frequency (20 Hz) TMS over a two‐week period (Grisaru, Chudakov, Yaroslavsky, & Belmaker, 1998). At post‐treatment, patients receiving right prefrontal TMS demonstrated significantly greater improvement in symptoms of mania (as evaluated via the YMRS (Young et al., 1978) and the CGI (Spearing et al., 1997)) relative to patients receiving left prefrontal TMS (Grisaru et al., 1998), thus paving the way for future studies of TMS in mania. Of note, the researchers stopped the study early as patients receiving left prefrontal TMS were demonstrating markedly low response despite being on stable pharmacological treatment. The authors concluded that left‐sided TMS may have prevented the action of anti‐manic pharmacotherapy (Grisaru et al., 1998; Kaptsan, Yaroslavsky, Applebaum, Belmaker, & Grisaru, 2003). As a follow‐up to their initial study, the authors randomly assigned patients (*n* = 19) to receive 10 sessions of right‐sided, high‐frequency (20 Hz) prefrontal TMS versus sham treatment over the course of two weeks. The authors found no difference between right‐sided TMS and sham TMS (Kaptsan et al., 2003), proposing the possibility that a more intensive treatment protocol is warranted for mania (e.g., greater treatment intensity or length; Kaptsan et al., 2003). One other study explored eight sessions of right prefrontal rapid TMS in bipolar patients experiencing a manic episode (*n* = 9) across a four‐week period. Patients experienced improvements in manic symptoms at post‐treatment (as evaluated by the Bech‐Rafaelsen mania scale (Bech, Rafaelsen, Kramp, & Bolwig, 1978)); however, this was an open‐label trial and thus cannot provide complete insight on the efficacy of a right‐sided standard TMS protocol (Michael & Erfurth, 2004).

The remaining studies of TMS in mania were rTMS protocols. Saba and colleagues conducted a pilot trial of 10‐session, high‐frequency (10 Hz) rTMS over the right DLPFC among patients with current mania (*n* = 8). After the two‐week treatment period, patients demonstrated a significant improvement in manic symptoms (as evaluated via the Mania Assessment Scale and CGI (Spearing et al., 1997)) (Saba et al., 2004). A subsequent trial randomized patients (*n* = 41) to receive 10 sessions of high‐frequency (20 Hz) rTMS over the right DLPFC or a sham treatment. Patients who received the active treatment demonstrated significant improvements in mania (as evaluated via the YMRS (Young et al., 1978)) relative to the sham group (Praharaj, Ram, & Arora, 2009). However, a follow‐up study employing an identical protocol in an adolescent sample found no significant differences in mania outcomes between the active and sham groups (Pathak, Sinha, & Praharaj, 2015). The authors suggest that the discrepant findings between the two studies may be accounted for by metabolic differences between adults and children. Specifically, adult patients with mania may have decreased metabolism on the right side of their brain and increased metabolism on the left side. Thus, in adults, an rTMS protocol over the right DLPFC may help account for these metabolic discrepancies. However, if adolescents do not exhibit this pattern of metabolic activity, they may not be as likely to respond to rTMS over their right DLPFC (Pathak et al., 2015).

To date, only one randomized study has suggested the potential benefit of a TMS protocol over the right DLPFC for mania, with that one study employing an rTMS protocol (Praharaj et al., 2009). It is possible that the repetitive nature of the magnetic pulses in the rTMS protocol yields a particular benefit for mania. However, a subsequent study that replicates the results from this positive trial in an adult sample is warranted to confirm that the failed rTMS trial in the adolescent sample was indeed due to different metabolic patterns in adolescents versus adults and not a broad sign of the treatment's limited efficacy.

### 3.3 TMS in other illness stages

A few open‐label studies have explored TMS across other phases of the bipolar illness. Li and colleagues evaluated TMS as a maintenance treatment in patients (*n* = 7) who had been successfully treated with TMS for their depression in a previously described study (Nahas et al., 2003). Patients received weekly maintenance TMS over the left prefrontal cortex for up to one year. Among the study patients, three continued with TMS for the full year and did not re‐enter an acute depressive episode during that period (Li, Nahas, Anderson, Kozel, & George, 2004). Another study explored 15 sessions of low‐frequency (1 Hz) rTMS over the right DLPFC for patients (*n* = 40) in a mixed bipolar episode. All patients also received a mood stabilizer as part of the study (e.g., valproate). At post‐treatment, the responder rate for depressive symptoms (as assessed via the HAM‐D (Hamilton, 1960)) was 46%, of which 29% met criteria for full remission, and the responder rate for manic symptoms (as assessed via the YMRS (Young et al., 1978)) was 15% with all meeting criteria for full remission (Pallanti et al., 2014). These positive trials suggest that future randomized studies may wish to evaluate TMS as a maintenance option or as an intervention for bipolar mixed states.

Please refer to Table 1 for a summary of the reviewed TMS clinical trials in BD.

**Table 1:** Summary of transcranial magnetic stimulation (TMS) clinical trials in bipolar disorder

Authors	Design	Mood episode	Sample size	Sessions (#)	Notable outcomes
Dolberg et al. (2002)	Randomized, controlled study (rTMS vs. sham)	Bipolar depression	20	20 (rTMS), 10 (sham)	Reductions in HAM‐D and Brief Psychiatric Rating Scale scores favoring rTMS group
Nahas et al. (2003)	Randomized, controlled study (left‐sided rTMS vs. sham)	Bipolar depression (two participants in a mixed state)	23	10	No significant between‐group differences, trend of improved subjective mood favoring rTMS group
Tamas et al. (2007)	Randomized, controlled study (right‐sided, low‐frequency rTMS vs. sham)	Bipolar depression	5	8	Reductions in HAM‐D scores favoring rTMS group
Fitzgerald, Benitez, et al. (2006)	Randomized study (right‐sided 1 Hz rTMS vs. right‐sided 2 Hz rTMS)	Bipolar depression	25	10+	Significant effect of time on treatment outcomes, nonsignificant trend toward greater improvements for 2 Hz over 1 Hz treatment
Dell'Osso et al. (2009), Dell'osso et al. (2011)	Open study (low‐frequency, right‐sided rTMS combined with magnetic resonance imaging)	Bipolar depression	11	15	Significant reductions in HAM‐D, MADRS, and CGI scores, acute treatment response predicted outcomes at 1 year
Dell'Osso et al. (2015)	Randomized study (low‐frequency, right‐sided rTMS with pauses vs. low‐frequency, right‐sided rTMS at continuous rate vs. high‐frequency, left‐sided rTMS with pauses)	Bipolar depression	33	20	Significant reductions in HAM‐D, MADRS, and CGI scores across all groups, no between‐group differences
Hu et al. (2016)	Randomized, controlled study (right‐sided, low‐frequency rTMS vs. left‐sided, high‐frequency rTMS vs. sham, all adjunctive to quetiapine)	Bipolar depression	38	20	Reductions in HAM‐D and MADRS scores during treatment, no between‐group differences
Fitzgerald, Benitez, et al. (2006)	Randomized, controlled study (right‐sided, low‐frequency followed by left‐sided, high‐frequency bilateral rTMS vs. sham)	Bipolar depression	8	10+	2 out of 4 patients in active group showed improvements compared to 1 out of 4 patients in sham group
Fitzgerald et al. (2016)	Randomized, controlled study (right‐sided, low‐frequency followed by left‐sided, high‐frequency bilateral rTMS vs. sham)	Bipolar depression	49	20	No between‐group differences
Carnell et al. (2017)	Randomized study (low‐frequency right‐sided rTMS vs. left‐sided, high‐frequency followed by right‐sided, low‐frequency bilateral rTMS)	Bipolar depression	50	18	Significant reductions in HAM‐D scores across all patients, no between‐group differences
Kazemi et al. (2016)	Randomized study (right‐sided, low‐frequency rTMS vs. right‐sided, low‐frequency followed by left‐sided, high‐frequency bilateral rTMS)	Bipolar depression	30	20	Significantly more responders in bilateral group (80% of patients) relative to unilateral group (47% of patients)
Harel et al. (2011)	Open study (high‐frequency deep rTMS)	Bipolar depression	19	20	Significant reductions in HAM‐D scores
Tavares et al. (2017)	Randomized, controlled study (left‐sided, high‐frequency deep rTMS vs. sham)	Bipolar depression	50	20	Significant reductions in HAM‐D scores at post‐treatment favoring rTMS (not maintained at follow‐up)
Rapinesi et al. (2018)	Open study (high‐frequency, bilateral deep rTMS)	Bipolar depression	20	20	80% of patients showed response following acute treatment
Beynel et al. (2014)	Open study (intermittent theta burst stimulation vs. sham treatment)	Bipolar depression	12	10+	Reductions in MADRS scores favoring theta burst stimulation group
Grisaru et al. (1998)	Randomized study (right‐sided, high‐frequency vs. left‐sided, high‐frequency TMS)	Mania	16	10	Greater reductions in YMRS and CGI scores favoring right‐sided group, notably low response in left‐sided group
Kaptsan et al. (2003)	Randomized, controlled study (right‐sided, high‐frequency TMS vs. sham)	Mania	19	10	No differences between groups
Michael and Erfurth (2004)	Open study (right‐sided, rapid TMS)	Mania	9	8	Reductions in Bech‐Rafaelsen scores
Saba et al. (2004)	Open study (right‐sided, high‐frequency rTMS)	Mania	8	10	Significant reductions in Mania Assessment Scale and CGI scores
Praharaj et al. (2009)	Randomized, controlled study (right‐sided, high‐frequency rTMS vs. sham)	Mania	41	10	Significant reductions in YMRS scores favoring active group
Pathak et al. (2015)	Randomized, controlled study (right‐sided, high‐frequency rTMS vs. sham)	Mania	26	10	No significant between‐group differences
Li et al. (2004)	Open study (left‐sided TMS)	No active episode upon study entry	7	50, 34, 46^a^	Three study patients did not experience relapse into acute depression following one year of TMS
Pallanti et al. (2014)	Open study (right‐sided, low‐frequency rTMS adjunctive to mood stabilizer)	Mixed	40	15	Reductions in HAM‐D and YMRS scores for some patients

Abbreviations: CGI, Clinical Global Impressions Scale (Spearing et al., 1997); HAM‐D, Hamilton Rating Scale for Depression (Hamilton, 1960); MADRS, Montgomery‐Asberg Depression Rating Scale (Montgomery & Asberg, 1979); TMS, transcranial magnetic stimulation.

a Number of TMS sessions completed by three study patients who completed one year of weekly TMS.

## 4 DISCUSSION

Transcranial magnetic stimulation represents an important, largely understudied avenue of intervention research and clinical care in BD. This review synthesizes data from the few clinical trials that have explored TMS as a treatment for patients with BD across varied mood stages. To date, most research has focused on rTMS for patients in a bipolar depressive episode. Perhaps for this reason, rTMS for bipolar depression has the greatest empirical support with several studies suggesting the treatment's potential in reducing depressive symptomatology (Beynel et al., 2014; Dell'Osso et al., 2009, 2015; Dolberg et al., 2002; Harel et al., 2011; Tamas et al., 2007), though studies are more varied in their findings on the most effective rTMS protocol (e.g., high‐frequency vs. low‐frequency, right‐sided vs. left‐sided, bilateral vs. unilateral). TMS for mania has been the focus of fewer clinical trials and yielded more inconsistent findings with only one randomized, controlled trial suggesting the benefit of rTMS over a sham treatment (Praharaj et al., 2009). Of note, despite the disparate study outcomes, nearly all the studies of TMS for mania targeted similar right prefrontal cortical regions. Open‐label studies of TMS for bipolar mixed states (Pallanti et al., 2014) and for maintenance care (Li et al., 2004) have also delivered promising findings.

There are some important limitations associated with existing clinical trials of TMS in BD. Among the few studies that have evaluated TMS as a treatment for BD, many are limited by small samples with most studies incorporating 20 or fewer patients. Thus, a challenge for upcoming research in TMS will be to conduct larger‐scale studies of TMS in BD with a focus on enhancing knowledge on specific TMS protocols (e.g., in selecting a TMS approach for a given bipolar depressed patient with a specific clinical profile, what protocol will likely be most effective?). Determining an answer to this question will also involve further clarifying the specific parameters that are applied in TMS protocols. For instance, many of the reviewed TMS studies applied low‐frequency stimulation at 1 Hz and high‐frequency stimulation at 10 Hz. However, a few studies applied different stimulation thresholds (such as 2 Hz; Fitzgerald, Huntsman, et al., 2006 and 20 Hz Grisaru et al., 1998; Harel et al., 2011; Rapinesi et al., 2018) though it is important to note that these studies did not all incorporate the same type of TMS treatment (e.g., some studies involved standard rTMS whereas other studies evaluated deep TMS). It will be necessary to further clarify the type of TMS treatment along with the optimal threshold of stimulation that will be most helpful for a given patient with BD, particularly as findings from one study found a trend toward disparate outcomes for two different low‐frequency stimulation protocols (Fitzgerald, Huntsman, et al., 2006).

Additional large‐scale studies will also aid in clarifying predictors of response to specific TMS protocols. A study that incorporated a large BD sample (*n* = 146) found that “cognitive–affective” (e.g., emotional or cognitive) as opposed to somatic (e.g., bodily related) symptoms of depression predicted a superior response to rTMS with loss of interest being the most significant cognitive–affective symptom influencing treatment response (Rostami, Kazemi, Nitsche, Gholipour, & Salehinejad, 2017). Another study found that patients (*n* = 30) with treatment‐resistant bipolar depression who were being treated with certain antidepressants (e.g., trazodone, mirtazapine, mianserin) or antihistamines (e.g., promethazine, hydroxyzine) or who had a longer illness duration had a poorer response to high‐frequency rTMS (Poleszczyk, Rakowicz, Parnowski, Antczak, & Święcicki, 2018). By contrast, patients in this study who reported more sleep disturbances demonstrated a superior response to high‐frequency rTMS (Poleszczyk et al., 2018). Thus, elucidating not only which illness characteristics affect TMS response but also *why* certain illness characteristics lead to a superior (or absence of) TMS response is key.

Recently, studies have suggested the potential benefit of TMS for cognition among patients with BD. Preliminary research suggests that TMS can be helpful for improving a range of cognitive functions as evaluated via neuropsychological assessments such as verbal fluency (Thomas‐Ollivier et al., 2017), immediate and delayed verbal memory (Kazemi et al., 2018; Myczkowski et al., 2018), executive functioning (Kazemi et al., 2018), working memory (Myczkowski et al., 2018), attention and processing speed (Myczkowski et al., 2018), and inhibitory control (Myczkowski et al., 2018). Notably, these few studies incorporated varied TMS protocols (bilateral vs. unilateral) and types of TMS treatment (deep TMS vs. standard rTMS), so it is not yet possible to definitively match specific TMS protocols to certain cognitive improvements. It will also be important to assess whether laboratory‐based cognitive gains map onto cognitive improvements in daily functioning at work and at home.

Other important considerations are worthy of note. First, in most of these trials, patients were receiving adjunctive pharmacotherapy. Thus, findings from these studies may not be entirely generalizable in that patients with BD have unique and complex medication regimens (Lin, Mok, & Yatham, 2006). Yet, this caveat is not so much a limitation as a reflection on these studies' capacity to reflect “real‐world” bipolar patients who may be interested in pursuing TMS treatment. Second, across the reviewed studies, patients experienced side effects from TMS treatment, though most of these were described as mild. The most common mild side effects among the studies of TMS for bipolar depression were headaches and insomnia with other side effects including local pain at the site of administration, fatigue, memory difficulties, and dizziness (Dell'Osso et al., 2009, 2015; Tamas et al., 2007; Tavares et al., 2017). Most notably, in three bipolar depression studies, patients experienced a switch into a manic episode either during or shortly after treatment (Dell'Osso et al., 2015; Dolberg, Schreiber, & Grunhaus, 2001) and, in one study, a patient with bipolar depression experienced transient hypomania after three weeks of left‐sided, high‐frequency rTMS (Hu et al., 2016).

It will be helpful for future studies to more clearly elucidate how clinicians can recognize risk factors for developing mania post‐TMS, enabling them to more effectively tailor their treatment for a given patient. Only two studies of TMS for mania noted that patients reported side effects; across these studies, patients experienced pain during their procedure (which went away after session completion), dizziness, anxiety, and a brief headache following treatment (Pathak et al., 2015; Praharaj et al., 2009). The trial evaluating TMS for a bipolar mixed state reported only minor side effects in a few patients that included headaches, insomnia, and pain at the site of administration (Pallanti et al., 2014), whereas the trial that studied TMS as a bipolar maintenance treatment reported no side effects (Li et al., 2004). Ultimately, the overall minor and noninterfering nature of most side effects represents another promising aspect of TMS treatment, potentially facilitating treatment adherence and engagement.

## CONFLICTS OF INTEREST

Ms. Gold receives research support from the National Institute of Mental Health (F31MH116557). Dr. Ornelas has no competing interests to report. Dr. Cirillo has no competing interest to report. Dr. Caldieraro has no competing interests to report. Dr. Nardi has no competing interests to report. Dr. Nierenberg reports the following disclosures: *Consultant—*Abbott Laboratories, Alkermes, American Psychiatric Association, Appliance Computing Inc. (Mindsite), Basliea, Brain Cells, Inc., Brandeis University, Bristol‐Myers Squibb, Clintara, Corcept, Dey Pharmaceuticals, Dainippon Sumitomo (now Sunovion), Eli Lilly and Company, EpiQ, L.P./Mylan Inc., Forest, Genaissance, Genentech, GlaxoSmithKline, Healthcare Global Village, Hoffman LaRoche, Infomedic, Intra‐Cellular Therapies, Lundbeck, Janssen Pharmaceutica, Jazz Pharmaceuticals, Medavante, Merck, Methylation Sciences, NeuroRx, Naurex, Novartis, PamLabs, Parexel, Pfizer, PGx Health, Otsuka, Ridge Diagnostics Shire, Schering‐Plough, Somerset, Sunovion, Takeda Pharmaceuticals, Targacept, and Teva**;** consulted through the MGH Clinical Trials Network and Institute (CTNI) for AstraZeneca, Brain Cells, Inc, Dianippon Sumitomo/Sepracor, Johnson and Johnson, Labopharm, Merck, Methylation Science, Novartis, PGx Health, Shire, Schering‐Plough, Targacept and Takeda/Lundbeck Pharmaceuticals, NeuroRx Pharma, Pfizer, Physician's Postgraduate Press, Inc. *Grants/Research support—*American Foundation for Suicide Prevention, AHRQ, Brain and Behavior Research Foundation, Bristol‐Myers Squibb, Cederroth, Cephalon, Cyberonics, Elan, Eli Lilly & Company, Forest, GlaxoSmithKline, Intra‐Cellular Therapies, Janssen Pharmaceuticals, Lichtwer Pharma, Marriott Foundation, Mylan, NIMH, PamLabs, Patient Centered Outcomes Research Institute (PCORI), Pfizer Pharmaceuticals, Shire, Stanley Foundation, Takeda/Lundbeck, and Wyeth‐Ayerst. *Honoraria—*Belvoir Publishing, University of Texas Southwestern Dallas, Brandeis University, Bristol‐Myers Squibb, Hillside Hospital, American Drug Utilization Review, American Society for Clinical Psychopharmacology, Baystate Medical Center, Columbia University, CRICO, Dartmouth Medical School, Health New England, Harold Grinspoon Charitable Foundation, IMEDEX, International Society for Bipolar Disorder, Israel Society for Biological Psychiatry, Johns Hopkins University, MJ Consulting, New York State, Medscape, MBL Publishing, MGH Psychiatry Academy, National Association of Continuing Education, Physicians Postgraduate Press, SUNY Buffalo, University of Wisconsin, University of Pisa, University of Michigan, University of Miami, University of Wisconsin at Madison, APSARD, ISBD, SciMed, Slack Publishing and Wolters Klower Publishing, ASCP, NCDEU, Rush Medical College, Yale University School of Medicine, NNDC, Nova Southeastern University, NAMI, Institute of Medicine, CME Institute, ISCTM, World Congress on Brain Behavior and Emotion, Congress of the Hellenic Society for Basic and Clinical Pharmacology, ADAA. *Stock* **—**Appliance Computing, Inc. (MindSite); Brain Cells, Inc., Medavante**.** *Copyrights*—Clinical Positive Affect Scale and the MGH Structured Clinical Interview for the Montgomery‐Asberg Depression Scale exclusively licensed to the MGH Clinical Trials Network and Institute (CTNI). *Speaker Bureaus*—none since 2003. Dr. Kinrys has received research support from AstraZeneca, Bristol‐Myers Squibb Company, Cephalon, Elan Pharmaceuticals, Eli Lilly & Company, Forest Pharmaceuticals Inc., GlaxoSmithkline, Sanofi/Synthelabo, Sepracor Inc., Pfizer Inc, UCB Pharma, and Wyeth‐Ayerst Laboratories, Agency for Healthcare Research and Quality (AHRQ) Grant R01 HS019371‐01, and Takeda Pharmaceuticals. He has been an advisor or consultant for AstraZeneca, Cephalon, Eli Lilly & Company, Forest Pharmaceuticals Inc., GlaxoSmithkline, Janssen Pharmaceutica, Pfizer Inc, Sepracor Inc., UCB Pharma, and Wyeth‐Ayerst Laboratories. Dr. Kinrys has been a speaker for AstraZeneca, Forest Pharmaceuticals Inc., GlaxoSmithkline, Sepracor Inc., and Wyeth‐Ayerst Laboratories.

## AUTHOR CONTRIBUTIONS

AKG, GK, ACO, PC, MAC, AEN, and AAN contributed to the writing and editing of this article. AKG, ACO, PC, and MAC were responsible for searching databases and articles for the relevant papers. All authors approved the final version of this article.

## Data Availability

Data sharing is not applicable to this article as no new data were created or analyzed in this study.

## References

[brb31419-bib-0001] Bech, P. , Rafaelsen, O. , Kramp, P. , & Bolwig, T. (1978). The mania rating scale: Scale construction and inter‐observer agreement. Neuropharmacology, 17(6), 430–431. 10.1016/0028-3908(78)90022-9 673161

[brb31419-bib-0002] Beynel, L. , Chauvin, A. , Guyader, N. , Harquel, S. , Szekely, D. , Bougerol, T. , & Marendaz, C. (2014). What saccadic eye movements tell us about TMS‐induced neuromodulation of the DLPFC and mood changes: A pilot study in bipolar disorders. Frontiers in Integrative Neuroscience, 8, 65 10.3389/fnint.2014.00065 25191234 PMC4137451

[brb31419-bib-0003] Brunelin, J. , Jalenques, I. , Trojak, B. , Attal, J. , Szekely, D. , Gay, A. , … Poulet, E. (2014). The efficacy and safety of low frequency repetitive transcranial magnetic stimulation for treatment‐resistant depression: The results from a large multicenter French RCT. Brain Stimulation, 7(6), 855–863. 10.1016/j.brs.2014.07.040 25192980

[brb31419-bib-0004] Bulteau, S. , Sébille, V. , Fayet, G. , Thomas‐Ollivier, V. , Deschamps, T. , Bonnin‐Rivalland, A. , … Sauvaget, A. (2017). Efficacy of intermittent Theta Burst Stimulation (iTBS) and 10‐Hz high‐frequency repetitive transcranial magnetic stimulation (rTMS) in treatment‐resistant unipolar depression: Study protocol for a randomised controlled trial. Trials, 18(1), 17 10.1186/s13063-016-1764-8 28086851 PMC5237321

[brb31419-bib-0005] Carnell, B. L. , Clarke, P. , Gill, S. , & Galletly, C. A. (2017). How effective is repetitive transcranial magnetic stimulation for bipolar depression? Journal of Affective Disorders, 209, 270–272. 10.1016/j.jad.2016.11.041 27987405

[brb31419-bib-0006] Cohen, R. B. , Brunoni, A. R. , Boggio, P. S. , & Fregni, F. (2010). Clinical predictors associated with duration of repetitive transcranial magnetic stimulation treatment for remission in bipolar depression: A naturalistic study. The Journal of Nervous and Mental Disease, 198(9), 679–681. 10.1097/NMD.0b013e3181ef2175 20823731

[brb31419-bib-0007] Connolly, K. R. , Helmer, A. , Cristancho, M. A. , Cristancho, P. , & O'Reardon, J. P. (2012). Effectiveness of transcranial magnetic stimulation in clinical practice post‐FDA approval in the United States: Results observed with the first 100 consecutive cases of depression at an academic medical center. Journal of Clinical Psychiatry, 73(4), e567–e573. 10.4088/JCP.11m07413 22579164

[brb31419-bib-0008] Dell'osso, B. , D'Urso, N. , Castellano, F. , Ciabatti, M. , & Altamura, A. C. (2011). Long‐term efficacy after acute augmentative repetitive transcranial magnetic stimulation in bipolar depression: A 1‐year follow‐up study. The Journal of ECT, 27(2), 141–144. 10.1097/YCT.0b013e3181f66601 20966770

[brb31419-bib-0009] Dell'Osso, B. , Mundo, E. , D'Urso, N. , Pozzoli, S. , Buoli, M. , Ciabatti, M. T. , … Carlo Altamura, A. (2009). Augmentative repetitive navigated transcranial magnetic stimulation (rTMS) in drug‐resistant bipolar depression. Bipolar Disorders, 11(1), 76–81. 10.1111/j.1399-5618.2008.00651.x 19133969

[brb31419-bib-0010] Dell'Osso, B. , Oldani, L. , Camuri, G. , Dobrea, C. , Cremaschi, L. , Benatti, B. , … Carlo Altamura, A. (2015). Augmentative repetitive Transcranial Magnetic Stimulation (rTMS) in the acute treatment of poor responder depressed patients: A comparison study between high and low frequency stimulation. European Psychiatry, 30(2), 271–276. 10.1016/j.eurpsy.2014.12.001 25572482

[brb31419-bib-0011] Dolberg, O. , Dannon, P. , Schreiber, S. , & Grunhaus, L. (2002). Transcranial magnetic stimulation in patients with bipolar depression: A double blind, controlled study. Bipolar Disorders, 4, 94–95. 10.1034/j.1399-5618.4.s1.41.x 12479689

[brb31419-bib-0012] Dolberg, O. T. , Schreiber, S. , & Grunhaus, L. (2001). Transcranial magnetic stimulation‐induced switch into mania: A report of two cases. Biological Psychiatry, 49(5), 468–470. 10.1016/S0006-3223(00)01086-6 11274660

[brb31419-bib-0013] Fitzgerald, P. B. , Benitez, J. , de Castella, A. , Daskalakis, Z. J. , Brown, T. L. , & Kulkarni, J. (2006). A randomized, controlled trial of sequential bilateral repetitive transcranial magnetic stimulation for treatment‐resistant depression. American Journal of Psychiatry, 163(1), 88–94. 10.1176/appi.ajp.163.1.88 16390894

[brb31419-bib-0014] Fitzgerald, P. B. , Hoy, K. E. , Elliot, D. , McQueen, S. , Wambeek, L. E. , & Daskalakis, Z. J. (2016). A negative double‐blind controlled trial of sequential bilateral rTMS in the treatment of bipolar depression. Journal of Affective Disorders, 198, 158–162. 10.1016/j.jad.2016.03.052 27016659

[brb31419-bib-0015] Fitzgerald, P. B. , Huntsman, S. , Gunewardene, R. , Kulkarni, J. , & Daskalakis, Z. J. (2006). A randomized trial of low‐frequency right‐prefrontal‐cortex transcranial magnetic stimulation as augmentation in treatment‐resistant major depression. International Journal of Neuropsychopharmacology, 9(6), 655–666. 10.1017/s1461145706007176 16959055

[brb31419-bib-0016] Fountoulakis, K. N. , Grunze, H. , Vieta, E. , Young, A. , Yatham, L. , Blier, P. , … Moeller, H. J. (2017). The International College of Neuro‐Psychopharmacology (CINP) treatment guidelines for Bipolar disorder in adults (CINP‐BD‐2017), part 3: The clinical guidelines. International Journal of Neuropsychopharmacology, 20(2), 180–195. 27941079 10.1093/ijnp/pyw109 PMC5408976

[brb31419-bib-0017] Geddes, J. R. , & Miklowitz, D. J. (2013). Treatment of bipolar disorder. The Lancet, 381(9878), 1672–1682. 10.1016/S0140-6736(13)60857-0 PMC3876031 23663953

[brb31419-bib-0018] George, M. S. , Nahas, Z. , Molloy, M. , Speer, A. M. , Oliver, N. C. , Li, X.‐B. , … Ballenger, J. C. (2000). A controlled trial of daily left prefrontal cortex TMS for treating depression. Biological Psychiatry, 48(10), 962–970. 10.1016/S0006-3223(00)01048-9 11082469

[brb31419-bib-0019] Grisaru, N. , Chudakov, B. , Yaroslavsky, Y. , & Belmaker, R. (1998). Transcranial magnetic stimulation in mania: A controlled study. American Journal of Psychiatry, 155(11), 1608–1610. 10.1176/ajp.155.11.1608 9812128

[brb31419-bib-0020] Hamilton, M. (1960). A rating scale for depression. Journal of Neurology, Neurosurgery and Psychiatry, 23, 56–62. 10.1136/jnnp.23.1.56 PMC495331 14399272

[brb31419-bib-0021] Harel, E. V. , Zangen, A. , Roth, Y. , Reti, I. , Braw, Y. , & Levkovitz, Y. (2011). H‐coil repetitive transcranial magnetic stimulation for the treatment of bipolar depression: An add‐on, safety and feasibility study. The World Journal of Biological Psychiatry, 12(2), 119–126. 10.3109/15622975.2010.510893 20854181

[brb31419-bib-0022] Hu, S.‐H. , Lai, J.‐B. , Xu, D.‐R. , Qi, H.‐L. , Peterson, B. S. , Bao, A.‐M. , … Xu, Y. I. (2016). Efficacy of repetitive transcranial magnetic stimulation with quetiapine in treating bipolar II depression: A randomized, double‐blinded, control study. Scientific Reports, 6, 30537 10.1038/srep30537 27460201 PMC4962310

[brb31419-bib-0023] Kaptsan, A. , Yaroslavsky, Y. , Applebaum, J. , Belmaker, R. , & Grisaru, N. (2003). Right prefrontal TMS versus sham treatment of mania: A controlled study. Bipolar Disorders, 5(1), 36–39. 10.1034/j.1399-5618.2003.00003.x 12656936

[brb31419-bib-0024] Kazemi, R. , Rostami, R. , Khomami, S. , Baghdadi, G. , Rezaei, M. , Hata, M. , Fitzgerald, P. B. (2018). Bilateral transcranial magnetic stimulation on DLPFC changes resting state networks and cognitive function in patients with bipolar depression. Frontiers in Human Neuroscience, 12, 1–10. 10.3389/fnhum.2018.00356 30233346 PMC6135217

[brb31419-bib-0025] Kazemi, R. , Rostami, R. , Khomami, S. , Horacek, J. , Brunovsky, M. , Novak, T. , & Fitzgerald, P. B. (2016). Electrophysiological correlates of bilateral and unilateral repetitive transcranial magnetic stimulation in patients with bipolar depression. Psychiatry Research, 240, 364–375. 10.1016/j.psychres.2016.04.061 27138833

[brb31419-bib-0026] Kemp, D. E. , Sylvia, L. G. , Calabrese, J. R. , Nierenberg, A. A. , Thase, M. E. , Reilly‐Harrington, N. A. , … Iosifescu, D. V. (2014). General medical burden in bipolar disorder: Findings from the LiTMUS comparative effectiveness trial. Acta Psychiatrica Scandinavica, 129(1), 24–34. 10.1111/acps.12101 23465084 PMC3789858

[brb31419-bib-0027] Li, X. , Nahas, Z. , Anderson, B. , Kozel, F. A. , & George, M. S. (2004). Can left prefrontal rTMS be used as a maintenance treatment for bipolar depression? Depress Anxiety, 20(2), 98–100. 10.1002/da.20027 15390210

[brb31419-bib-0028] Lin, D. , Mok, H. , & Yatham, L. N. (2006). Polytherapy in bipolar disorder. CNS Drugs, 20(1), 29–42. 10.2165/00023210-200620010-00003 16396522

[brb31419-bib-0029] Loo, C. K. , & Mitchell, P. B. (2005). A review of the efficacy of transcranial magnetic stimulation (TMS) treatment for depression, and current and future strategies to optimize efficacy. Journal of Affective Disorders, 88(3), 255–267. 10.1016/j.jad.2005.08.001 16139895

[brb31419-bib-0030] Martin, L. R. , Williams, S. L. , Haskard, K. B. , & Dimatteo, M. R. (2005). The challenge of patient adherence. Therapeutics and Clinical Risk Management, 1(3), 189–199. 18360559 PMC1661624

[brb31419-bib-0031] Matson, J. L. , González, M. L. , Smith, K. R. , Terlonge, C. , Thorson, R. T. , & Dixon, D. R. (2006). Assessing side effects of pharmacotherapy treatment of bipolar disorder: A 20‐year review of the literature. Research in Developmental Disabilities, 27(5), 467–500. 10.1016/j.ridd.2005.06.002 16143494

[brb31419-bib-0032] Michael, N. , & Erfurth, A. (2004). Treatment of bipolar mania with right prefrontal rapid transcranial magnetic stimulation. Journal of Affective Disorders, 78(3), 253–257. 10.1016/S0165-0327(02)00308-7 15013251

[brb31419-bib-0033] Mishra, B. R. , Sarkar, S. , Praharaj, S. K. , Mehta, V. S. , Diwedi, S. , & Nizamie, S. H. (2011). Repetitive transcranial magnetic stimulation in psychiatry. Annals of Indian Academy of Neurology, 14(4), 245 10.4103/0972-2327.91935 22346010 PMC3271460

[brb31419-bib-0034] Montgomery, S. A. , & Asberg, M. (1979). A new depression scale designed to be sensitive to change. British Journal of Psychiatry, 134, 382–389. 10.1192/bjp.134.4.382 444788

[brb31419-bib-0035] Myczkowski, M. L. , Fernandes, A. , Moreno, M. , Valiengo, L. , Lafer, B. , Moreno, R. A. , … Brunoni, A. R. (2018). Cognitive outcomes of TMS treatment in bipolar depression: Safety data from a randomized controlled trial. Journal of Affective Disorders, 235, 20–26. 10.1016/j.jad.2018.04.022 29631203

[brb31419-bib-0036] Nahas, Z. , Kozel, F. A. , Li, X. , Anderson, B. , & George, M. S. (2003). Left prefrontal transcranial magnetic stimulation (TMS) treatment of depression in bipolar affective disorder: A pilot study of acute safety and efficacy. Bipolar Disorders, 5(1), 40–47. 10.1034/j.1399-5618.2003.00011.x 12656937

[brb31419-bib-0037] Overall, J. E. , & Gorham, D. R. (1962). The brief psychiatric rating scale. Psychological Reports, 10(3), 799–812. 10.2466/pr0.1962.10.3.799

[brb31419-bib-0038] Özerdem, A. , Güntekin, B. , Tunca, Z. , & Başar, E. (2008). Brain oscillatory responses in patients with bipolar disorder manic episode before and after valproate treatment. Brain Research, 1235, 98–108. 10.1016/j.brainres.2008.06.101 18644356

[brb31419-bib-0039] Pallanti, S. , Grassi, G. , Antonini, S. , Quercioli, L. , Salvadori, E. , & Hollander, E. (2014). rTMS in resistant mixed states: An exploratory study. Journal of Affective Disorders, 157, 66–71. 10.1016/j.jad.2013.12.024 24581830

[brb31419-bib-0040] Pathak, V. , Sinha, V. K. , & Praharaj, S. K. (2015). Efficacy of adjunctive high frequency repetitive transcranial magnetic stimulation of right prefrontal cortex in adolescent mania: A Randomized Sham‐Controlled Study. Clinical Psychopharmacology and Neuroscience, 13(3), 245–249. 10.9758/cpn.2015.13.3.245 26598581 PMC4662165

[brb31419-bib-0041] Poleszczyk, A. , Rakowicz, M. , Parnowski, T. , Antczak, J. , & Święcicki, Ł. (2018). Are there clinical and neurophysiologic predictive factors for a positive response to HF‐rTMS in patients with treatment‐resistant depression? Psychiatry Research, 264, 175–181. 10.1016/j.psychres.2018.03.084 29649674

[brb31419-bib-0042] Praharaj, S. K. , Ram, D. , & Arora, M. (2009). Efficacy of high frequency (rapid) suprathreshold repetitive transcranial magnetic stimulation of right prefrontal cortex in bipolar mania: A randomized sham controlled study. Journal of Affective Disorders, 117(3), 146–150. 10.1016/j.jad.2008.12.020 19178948

[brb31419-bib-0043] Rapinesi, C. , Kotzalidis, G. D. , Ferracuti, S. , Girardi, N. , Zangen, A. , Sani, G. , … Del Casale, A. (2018). Add‐on high frequency deep transcranial magnetic stimulation (dTMS) to bilateral prefrontal cortex in depressive episodes of patients with major depressive disorder, bipolar disorder I, and major depressive with alcohol use disorders. Neuroscience Letters, 671, 128–132. 10.1016/j.neulet.2018.02.029 29454034

[brb31419-bib-0044] Rostami, R. , Kazemi, R. , Nitsche, M. A. , Gholipour, F. , & Salehinejad, M. (2017). Clinical and demographic predictors of response to rTMS treatment in unipolar and bipolar depressive disorders. Clinical Neurophysiology, 128(10), 1961–1970. 10.1016/j.clinph.2017.07.395 28829979

[brb31419-bib-0045] Saba, G. , Rocamora, J. F. , Kalalou, K. , Benadhira, R. , Plaze, M. , Lipski, H. , & Januel, D. (2004). Repetitive transcranial magnetic stimulation as an add‐on therapy in the treatment of mania: A case series of eight patients. Psychiatry Research, 128(2), 199–202. 10.1016/j.psychres.2004.05.019 15488963

[brb31419-bib-0046] Shah, N. , Grover, S. , & Rao, G. P. (2017). Clinical practice guidelines for management of bipolar disorder. Indian Journal of Psychiatry, 59(Suppl 1), S51 10.4103/0019-5545.196974 28216785 PMC5310104

[brb31419-bib-0047] Spearing, M. K. , Post, R. M. , Leverich, G. S. , Brandt, D. , & Nolen, W. (1997). Modification of the Clinical Global Impressions (CGI) Scale for use in bipolar illness (BP): The CGI‐BP. Psychiatry Research, 73(3), 159–171. 10.1016/S0165-1781(97)00123-6 9481807

[brb31419-bib-0048] Tamas, R. L. , Menkes, D. , & El‐Mallakh, R. S. (2007). Stimulating research: A prospective, randomized, double‐blind, sham‐controlled study of slow transcranial magnetic stimulation in depressed bipolar patients. Journal of Neuropsychiatry and Clinical Neurosciences, 19(2), 198–199. 10.1176/jnp.2007.19.2.198 17431073

[brb31419-bib-0049] Tavares, D. F. , Myczkowski, M. L. , Alberto, R. L. , Valiengo, L. , Rios, R. M. , Gordon, P. , … Brunoni, A. R. (2017). Treatment of bipolar depression with deep TMS: Results from a double‐blind, randomized, parallel group. Sham‐Controlled Clinical Trial. Neuropsychopharmacology, 42(13), 2593–2601. 10.1038/npp.2017.26 28145409 PMC5686495

[brb31419-bib-0050] Thomas‐Ollivier, V. , Foyer, E. , Bulteau, S. , Pichot, A. , Valriviere, P. , Sauvaget, A. , & Deschamps, T. (2017). Cognitive component of psychomotor retardation in unipolar and bipolar depression: I s verbal fluency a relevant marker? Impact of repetitive transcranial stimulation. Psychiatry and Clinical Neurosciences, 71(9), 612–623. 10.1111/pcn.12529 28419623

[brb31419-bib-0051] Young, R. , Biggs, J. , Ziegler, V. , & Meyer, D. (1978). A rating scale for mania: Reliability, validity and sensitivity. British Journal of Psychiatry, 133, 429–435. 10.1192/bjp.133.5.429 728692

